# The roles of IL-19 and IL-20 in the inflammation of degenerative lumbar spondylolisthesis

**DOI:** 10.1186/s12950-018-0195-6

**Published:** 2018-09-18

**Authors:** Kuo-Yuan Huang, Yu-Hsiang Hsu, Wei-Yu Chen, Hui-Ling Tsai, Jing-Jou Yan, Jung-Der Wang, Wen-Lung Liu, Ruey-Mo Lin

**Affiliations:** 1 Department of Orthopedics, National Cheng Kung University Hospital, College of Medicine, National Cheng Kung University, Tainan, Taiwan; 2 Institute of Clinical Medicine, College of Medicine, National Cheng Kung University, Tainan, Taiwan; 3 Clinical Medicine Research Center, National Cheng Kung University Hospital, College of Medicine, National Cheng Kung University, Tainan, Taiwan; 4 Institute for Translational Research in Biomedicine, Kaohsiung Chang Gung, Memorial Hospital, Kaohsiung, Taiwan; 5 Department of Pathology, National Cheng Kung University Hospital, College of Medicine, National Cheng Kung University, Tainan, Taiwan; 6 Department of Public of Health, College of Medicine, National Cheng Kung University, Tainan, Taiwan; 7 Department of Orthopedics, Tainan Municipal An-Nan Hospital-China Medical University, Tainan, Taiwan

**Keywords:** IL-19, IL-20, Degenerative lumbar Spondylolisthesis, Inflammation

## Abstract

**Background:**

Degenerative lumbar spondylolisthesis (DLS) is a major cause of spinal canal stenosis and is often related to lower back pain. IL-20 is emerging as a potent angiogenic, chemotactic, and proinflammatory cytokine related to several chronic inflammatory bone disorders likes intervertebral disc herniation, rheumatoid arthritis (RA), osteoporosis, and bone fracture. IL-19 also acts as a proinflammatory cytokine in RA. The aim of the present study was to investigate whether IL-19 and IL-20 are involved in DLS and compare three different tissues including disc, facet joint, and ligamentum flavum of patients with DLS to verify which tissue is affected more by inflammation.

**Methods:**

Disc, facet joint and ligamentum flavum from 13 patients with DLS was retrieved, and the expression pattern of IL-19, IL-20, IL-20R1, IL-20R2, TNF-α, IL-1β, and MCP-1 was evaluated using immunohistochemical staining with specific antibodies. The disc cells were isolated and incubated with IL-19 and IL-20 under CoCl_2_-mimicked hypoxic conditions to analyze the proinflammatory cytokine expression pattern using real-time quantitative PCR with specific primers.

**Results:**

IL-19 and IL-20 were positively stained and accompanied by abundant expression of TNF-α, IL-1β, and MCP-1 in facet joints of DLS patients. IL-19 and IL-20’s receptors (IL-20R1 and IL-20R2) were expressed on chondrocytes and fibrocytes/fibroblasts in facet joint and ligamentum flavum tissues from patients with DLS. There was a significant correlation between the expression of IL-20 and IL-1β in facet joint. In vitro assay, IL-19 and IL-20 upregulated the expression of IL-1β, IL-6, TNF-α, IL-8, VEGF, and MCP-1 in primary cultured DLS disc cells under CoCl_2_-mimicked hypoxic conditions.

**Conclusions:**

IL-19, IL-20, and their receptors as well as proinflammatory cytokines (TNF-α, IL-1β, and MCP-1) were expressed more in facet joints than the other tissues in patients with DLS; therefore, the etiology of inflammation might be more facet-centric. IL-19 and IL-20 induced proinflammatory cytokine expression in disc cells and might play a role in the pathogenesis of DLS.

## Background

Intervertebral disc herniation (HIVD) and spinal degeneration caused by inflammatory reactions and the mechanical compression of neural tissue, were the major causes of lower back pain, sciatica, and disability that affect millions of people each year [1–3]. Spinal degeneration mainly includes spinal stenosis, degenerative spondylolisthesis, and degenerative scoliosis. Degenerative lumbar spondylolisthesis (DLS) usually occurs at the L4/L5 level, resulting from the incompetence of disc or failed facet-joint locking mechanism, or buckling ligamentum flavum after spine degenerates to some extent [2]. Lower back pain, sciatica and intermittent claudication are common symptoms, which results in disability in patients with DLS [1, 3, 4].

The intervertebral disc (IVD) is the largest avascular organ in the human body, and the metabolic exchange is predominantly reliant on the diffusion effect across cartilage endplate in the mature IVD [5, 6]. Blood vessels in the adjacent vertebral bones do not reach the inner component of the discs but end at the interface between IVD and the vertebrae [7]. As an avascular tissue, IVD remains in a hypoxic microenvironment [8]. In the presence of low oxygen tension, low pH, and low levels of glucose, most energy for the nucleus pulposus is derived from anaerobic glycolysis rather than from oxidative phosphorylation [9]. Previous studies [10–12] also indicated that hypoxia-inducible factors (HIF-1) are expressed in this tissue and play a role during intervertebral disc degeneration.

The etiology of DLS is multi-factorial, and includes spinal instability, neurological compromise, and inflammation [13, 14]. Such causative factors influence each other in the pathogenesis of DLS. The symptoms are intermittent, which indicates the existence of many episodes of inflammatory cascades, repeated injuries and repair. The degenerative structures of DLS include disc, facet joint, and ligamentum flavum; however, there is still lack of researches to investigate the inflammation of the three structures simultaneously in DLS, and to elucidate which structure is affected more by inflammation in DLS patients.

IL-19 and IL-20 are members of IL-10 family (IL-19, IL-20, IL-22, IL-24, and IL-26) [15–19] and share structural and limited sequence homology with IL-10, an important pleiotropic immune-regulatory cytokine and an anti-inflammatory cytokine [20, 21]. They are involved in various inflammatory diseases [22–25]. IL-19 and IL-20 act through a heterodimer receptor complex that consists of the IL-20R1 and IL-20R2. IL-20 is additionally able to signal through a second heterodimer receptor complex (IL-22R1/IL-20R2) [26]. We previously reported that IL-20 induced proinflammatory, chemotactic, angiogenetic, and matrix degradative responses in the intervertebral disc herniation, suggesting IL-20 plays a critical role in the pathogenesis of disc herniation [22]. Recent studies demonstrated that blocking of IL-19 or IL-20 reduced bone loss and ameliorated collagen-induced arthritis in rat, suggesting that IL-19 and IL-20 are important proinflammatory molecules in bone or joint related diseases [23–25, 27]. However, the expressions of IL-19 and IL-20 in degenerative spinal tissues of disc, facet joint, and ligamentum flavum of DLS are still unknown. Therefore, we designed a study using an immunohistochemical staining to elucidate whether IL-19, IL-20, and their receptors are expressed in degenerative disc, facet joint, and ligamentum flavum tissue in elderly patients with DLS and to investigate which degenerative structures are involved more in the inflammatory process of DLS including discs, facet joints, and ligamentum flavum. We also compared the expression of IL-19, IL-20, and their receptors between the disc tissues of elderly patients with DLS and adult patients with HIVD (the data of disc samples from HIVD patients came from [22]) to understand the differences of inflammation expression between these two different diseases, and age groups. Better understanding of the distribution of IL-19 and IL-20 among different degenerative spinal tissues (disc, facet joint, and ligamentum flavum) of DLS may improve future treatment modalities for patients with DLS.

## Methods

### Reagents and antibodies

The antibodies of IL-19, IL-20, IL-20R1, and IL-20R2, and IL-1β monoclonal antibodies were purchased from R&D Systems, Minneapolis, MN. Monocyte chemotactic protein (MCP)-1and tumor necrosis factor (TNF)-α were purchased from Sigma-Aldrich Co., St Louis, MO.

### Patients

Thirteen consecutive patients (4 men; 9 women; average age: 68 years old) who had been diagnosed with DLS of L4–5 and undergone posterior spinal surgery of L4–5 including posterior decompression, posterior instrumentation, and posterior lateral fusion with or without posterior lumbar interbody fusion in National Cheng Kung University Hospital were enrolled in this study. The written informed consents for participation in the study were obtained from participants, and no participants are children. All the procedures were approved by the Human Experiment and Ethics Committee of National Cheng Kung University Medical Center (IRB approval: ER-96-163.), and were done in accordance with the Guidelines of the Declaration of Helsinki. The DLS diagnosis was based on the clinical presentation of lower back pain with sciatica or intermittent claudication and a radiograph showing slippage of theL4–5 lumbar vertebrae; Meyerding grades 1 or 2, with the degenerative changes of disc space narrowing; facet joint hypertrophy; and endplate spurs [2, 14]. All patients received MRI examination, which revealed degenerated discs (one patient with Grade II, four patients with Grade III, six patients with Grade IV, and one patient with Grade V based on Pfirrmann’s criteria [28]), and hypertrophic facet joints (eight patients with bright signal on T2 weighted image), and buckling of ligamentum flavum as well as lateral recess or central stenosis over L4–5 level. Surgical indications included progressive motor deficit, cauda equina syndrome, intermittent claudication, mechanical back pain or persistent sciatica of L5 after failed conservative treatment of more than three months. We collected the surgical specimens of degenerated intervertebral disc, facet joint, and ligamentum flavum tissue from the thirteen elderly patients with DLS receiving posterior spinal surgery. A block of L4–5 facet joint (symptomatic side) along with the capsule and synovial tissue was harvested using a bone chisel, ligamentum flavum of L4–5 harvested using a curved curette, and intervertebral disc of L4–5 harvested using a disc rongeur. A half retrieved disc tissues were used for immunohistochemical staining, and another half disc tissues were collected for isolation of disc cells.

### Immunohistochemical staining

Immunohistochemically (IHC) staining with the monoclonal antibodies for IL-1β, TNF-α, MCP-1, IL-19, IL-20, IL-20R1, and IL-20R2 were investigated to analyze the expression of these cytokines and chemokines and the procedures was briefly described as below. Tissue samples were rinsed twice in phosphate-buffered saline (PBS), stored overnight in 3.7% formaldehyde, dehydrated and then embedded in paraffin. Paraffin-embedded sections were then deparaffinized, rehydrated and antigen-retrieval with Tris-EDTA buffer (10 mmol/L Tris Base, 1 mmol/L EDTA, 0.05% Tween-20, pH 9.0) at 95–100 °C for 30 min for immunohistochemistry staining. Sections were then incubated with 3% H_2_O_2_ to block intrinsic peroxidase, after washing, the sections were incubated with blocking reagent for 30 min and then with the monoclonal antibodies against IL-19, IL-20, IL-20R1, and IL-20R2 (as described previously [29]), TNF-α (1:50 diluted), IL-1β (1:100 diluted), and MCP-1(1:20 diluted) at 4 °C for overnight. Isotype mouse IgG_1_ was used as the negative control. After washing thrice with PBST (PBS-0.05% Tween-20), sections were then incubated with HRP conjugated secondary antibody for 40 min at room temperature. After washing thrice with PBST, DAB chromogen was incubated with sections at room temperature for 2–10 min (ImmPACT™ DAB, Vector Laboratories, Burlingame, CA USA). And then counter stained with hematoxylin (Merck, Darmstadt, Germany). After washing with running tap water, the sections were then dehydrated and then cleared in xylene and mounted with mounting medium (Entellan®, Merck, Darmstadt, Germany). At least two sections from each patient’s specimen were analyzed for IHC staining. The whole section from each patient’s specimen was evaluated for each case. At least 100 cells for each cell type were counted. Specific cell types were deemed positive if > 1% of the cells were stained. The immunostained sections were evaluated by two senior pathologists in an observer-blinded fashion.

### Primary culture of human intervertebral disc cells from the DLS patients

The human IVD cells at the L4–5 level were isolated from a half of degenerated disc tissues after the surgery of posterior lumbar interbody fusion in patients with DLS. The primary cultures of IVD cells from the degenerated disc were collected as described [22, 30] for further in vitro study. The disc tissues were digested with 0.2% collagenase type II (Worthington, Lakewood, NJ) in Dulbecco modified Eagle medium and Ham F-12 medium (DMEM/F12) (Gibco, Grand Island, NY) for 6 h. After enzymatic digestion, the suspension was centrifuged and washed with medium. The isolated IVD cells were cultured in serum-free DMEM/F12 medium, 100 U/ml penicillin, 100 μg/ml streptomycin, and 2 mmol/L l-glutamine (Gibco, Grand Island, NY).

### Real-time quantitative PCR

To mimic the hypoxic condition for elucidating the in vitro effect of IL-19 or IL-20 on the disc cells, primarily isolated IVD cells (3 × 10^5^ cells/well) were cultured in serum-free DMEM/F12 medium and incubated with IL-19 or IL-20 under CoCl_2_-mimicked hypoxic condition for 6 h. CoCl_2_ only (hypoxic condition control), and PBS only (normoxic condition control) as the control groups. Total RNA was extracted using Trizol reagent (Life Technologies, Carlsbad, CA) and underwent reverse transcription using SuperScript III (Invitrogen) according to the manufacturer’s instructions. The expression of mRNA was analyzed using quantitative PCR with gene-specific primers for IL-1β, IL-6, IL-8, TNF-α, VEGF, and MCP-1. Detection of amplified template was accomplished with SYBR Green I (Applied Biosystems) chemistry using a StepOnePlus detection system. Individual PCR products were analyzed using melting point analysis. Samples were heated from 50 to 95 °C, and the decline in fluorescent signals of each individual sample were assessed. The fluorescence/time-dependent generation of signals was assessed using the manufacturer’s software program. For calculation of the relative expression, we normalized to the gene encoding glyceraldehyde-3-phosphate dehydrogenase (GAPDH) and compared it with the mean control values.

### Statistical analysis

All data are expressed as mean ± standard error (SD). Prism 6.0 (GraphPad Software) and Microsoft Excel (Microsoft Inc) were used for the statistical analysis. For the IHC staining, we compared the mutual expression of IL-19, IL-20, TNF-α, IL-1β, and MCP-1using the Fisher exact test. For statistics of quantitative PCR data, ANOVA was used to compare the data between groups. Statistical significance was set at *P* < 0.05.

## Results

### Expression of IL-19 and IL-20 in the facet joint of patients with DLS

**Fig. 1:**
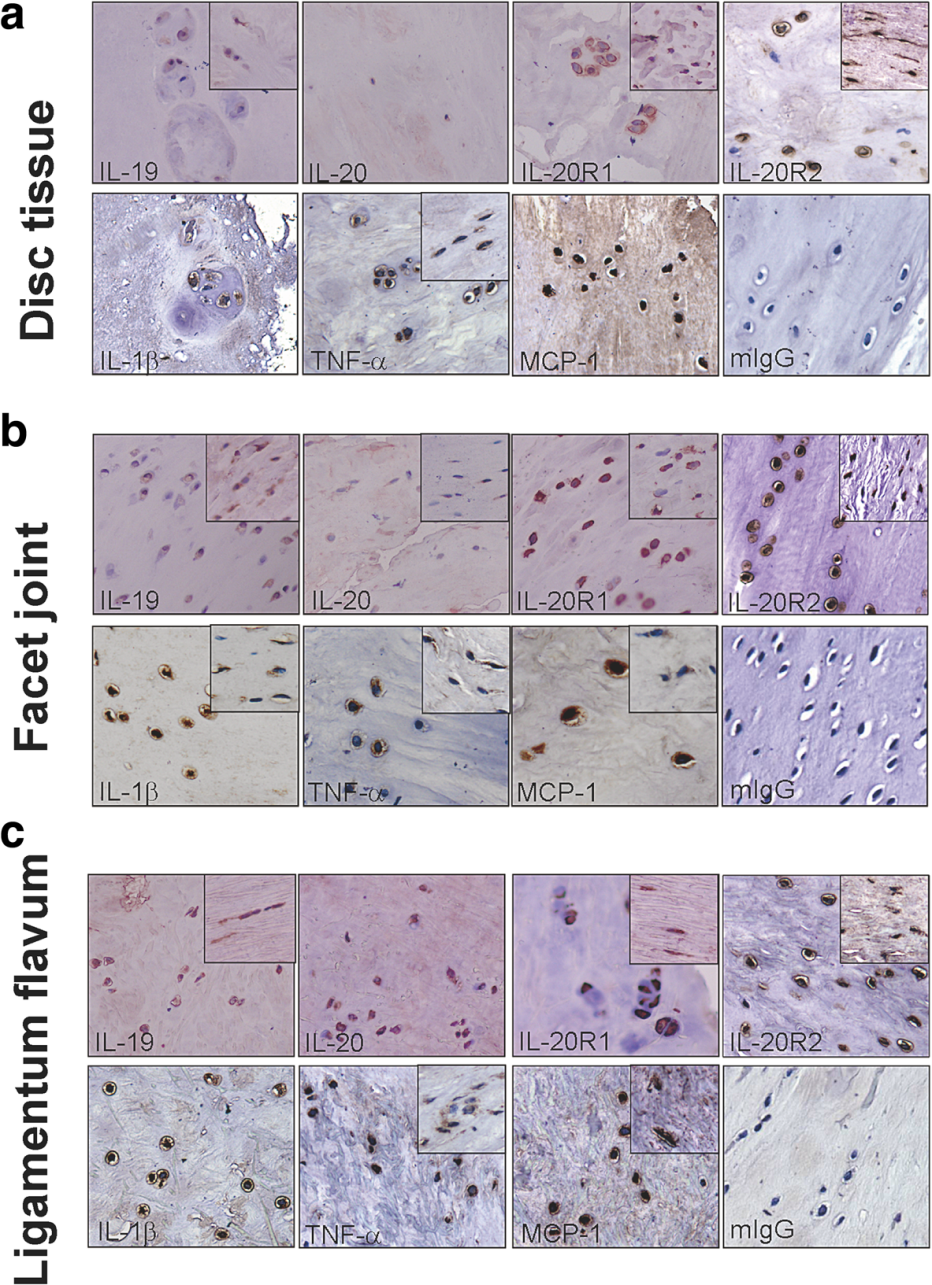
Expression of IL-19, IL-20, IL-20R1, IL-20R2, IL-1β, TNF-α, and MCP-1 in DLS patients. Micrographs of IHC staining of cytokines and chemokines (IL-19, IL-20, IL-20R1, IL-20R2, IL-1β, TNF-α, and MCP-1) with specific antibodies on (**a**) Disc, (**b**) Facet joint, and (**c**) Ligamentum flavum tissue samples from patients with DLS (Disc, *N* = 6; Facet joint, *N* = 12; Ligamentum flavum, *N* = 13). Chondrocytes were positively stained. The boxed area shows positively stained fibrocytes and fibroblasts (original magnification ×200). Staining with isotype mouse IgG was used as the negative control. All experiments were performed three times with similar results. Data are from a representative experiment.

To explore whether IL-19 and IL-20 are involved in the pathogenesis of DLS, we investigated the expression profile of IL-19, IL-20, IL-20R1, and IL-20R2 in different degenerated tissues of disc, facet joint, and ligamentum flavum from patients with DLS using IHC staining with specific antibodies (Fig. 1). In this study, it is difficult for us to quantify the cytokine levels in IHC staining because there are different cells in the degenerated tissue of facet joint, including fibroblasts, fibrocytes, chondrocytes, and synoviocytes; therefore, if there were positively stained cells in tissue specimen of the patients with DLS, the result of the IHC staining was categorized to have positive staining. To identify the specific sources of the cytokines, we mainly analyzed the immune reactivity of those cytokines and receptors in chondrocytes and fibrocytes/fibroblast in facet joint tissue, and both disc and ligamentum flavum tissues. We found that IL-19, IL-20, and receptors of IL-19 and IL-20 (IL-20R1 and IL-20R2) were positively stained in three different tissues, including disc, facet joint, and ligamentum flavum tissues from patients with DLS (Fig. 1 and Table1). To further clarify whether IL-19/IL-20 expression pattern is associated the other proinflammatory cytokines in DLS, we also analyze the expression pattern of three critical proinflammatory cytokines (IL-1β, TNF-α, and MCP-1) using IHC staining with specific antibodies. The ratios of positive immune reactivity of IL-19, IL-20, IL-1β, TNF-α, MCP-1, IL-20R1, and IL-20R2 in the discs, facet joints, and ligamentum flavum of patients with DLS was analyzed and summarized in Table 1.

**Table 1:** The results of immunohistochemical staining for inflammatory cytokines IL-19, IL-20, TNF-α, IL-1β, and MCP-1 and the receptors of IL-19 and IL-20 in disc, facet joint and ligamentum flavum tissues from patients with DLS

Number of Patients	Tissue type	IL-19		IL-20		IL-1β		TNF-α		MCP-1		IL-20R1		IL-20R2	
		Chon	Fibro	Chon	Fibro	Chon	Fibro	Chon	Fibro	Chon	Fibro	Chon	Fibro	Chon	Fibro
N = 6	D	3/6^a^	1/6	1/6	0/6	1/6	0/6	3/6	2/6	3/6	1/6	6/6	2/6	6/6	3/6
N = 12	F	6/12	6/12	5/12	8/12	8/12	2/12	10/12	10/12	9/12	5/12	12/12	8/12	12/12	12/12
N = 13	L	6/13	3/13	3/13	3/13	5/13	0/13	6/13	7/13	6/13	2/13	12/13	10/13	12/13	13/13

Abbreviation: *D* Disk, *F* Facet joint, *L* Ligamentum flavum, *Chon*. Chondrocytes, *Fibro*. Fibrocytes/Fibroblasts

^a^Ratio of positive staining for the specific cytokines in condrocytes or fibroblasts in tissue sections

In the discs of DLS patients, IL-19 was detected in chondrocytes in 3 of 6 samples (50%) and fibrocytes/fibroblasts in 1 of 6 samples (16.7%). IL-20R1 and IL-20R2 were stained in the chondrocytes in 6 of 6 disc tissues (100%), whereas IL-20 was only stained in chondrocytes in one disc tissue (1/6, 16.7%). TNF-α and MCP-1 were detected in chondrocytes in 3 of 6 samples (50%) and in fibrocytes/fibroblasts (2/6, and 1/6, respectively). IL-1β was only detected in 1 of 6 disc samples in chondrocytes (16.7%, Table 1).

In the facet joints of DLS patients, IL-19 and IL-20 were detected in chondrocytes in the facet joint tissues (6/12 and 5/12, respectively). IL-20R1 and IL-20R2 were generally detected in chondrocytes (12/12 and 12/12, respectively) and fibrocytes/fibroblasts (8/12 and 12/12, respectively) in facet joint tissues. TNF-α, MCP-1, and IL-1β were also detected in chondrocytes and fibrocytes/fibroblasts of the facet joint tissue. IL-1β was expressed in a higher ratio in facet joint tissue than in disc tissue (66.7% versus 16.7%, respectively). IL-1β was mainly detected in the chondrocytes (8/12, 66.7%) in comparison with fibrocytes/fibroblasts (2/12, 16.7%). TNF-α was detected in chondrocytes (10/12, 83.3%) and in fibrocytes/fibroblasts (10/12, 83.3%). MCP-1 was detected in the chondrocytes (9/12, 75%) and fibrocytes/fibroblasts (5/12, 41.7%) in the facet joint tissues (Table 1 and Fig. 1b).

In the ligamentum flavum of DLS patients, IL-19 and IL-20 were positively stained in chondrocytes (6/13 and 3/13, respectively) and fibrocytes/fibroblasts (3/13 and 3/13, respectively) in the ligamentum flavum tissues. IL-20R1 and IL-20R2 were generally detected in chondrocyte (12/13 and 12/13, respectively) and fibrocytes/fibroblasts (10/13 and 13/13, respectively). TNF-α and MCP-1were stained in chondrocytes (6/13 and 6/13, respectively) and fibrocytes/fibroblasts (7/13 and 2/13, respectively). IL-1β was only detected in chondrocytes (5/13) but not in fibrocytes/fibroblasts (0/13) (Table 1 and Fig. 1c).

According to the results of IHC staining of degenerated tissues of DLS, IL-19 and IL-20 were increased staining intensity and accompanied by abundant expression of TNF-α, IL-1β, and MCP-1 in facet joints of DLS patients except the equal expression of IL-19 in chondrocytes of disc tissues. Interestingly, IL-19 and IL-20’s receptors (IL-20R1 and IL-20R2) were expressed on chondrocytes and fibrocytes/fibroblasts in the disc, facet joint, and ligamentum flavum tissues from patients with DLS.

### IL-20 expression is positively correlated with IL-1β in facet joints of patients with DLS

To further clarify the association of IL-19/IL-20 expression pattern with other proinflammatory cytokines TNF-α, IL-1β, and MCP-1 in degenerated tissues of DLS, we used correlative analyses and found that the expression of IL-20 and IL-1β in facet joint was significantly correlated (*P* = 0.018, Table 2), whereas none of the cytokine expression was significantly correlated in disc and ligamentum flavum.

**Table 2:** Correlative analyses of cytokine expressions in facet joint tissues from DLS patients

Variables	IL-19 +	IL-20 +	IL-20R1 +	TNF-α +	IL-1β +	MCP-1 +
IL-19						
+		5 (83)	6 (100)	6 (100)	5 (83)	5 (83)
-		4 (67)	6 (100)	4 (67)	3 (50)	5 (83)
***P*** **-value**		1.000	–	0.455	0.546	1.000
IL-20						
+			9 (100)	8 (89)	8 (89)	7 (78)
-			3 (100)	2 (67)	0 (0)	3 (100)
***P*** **-value**			–	0.455	0.018*	1.000
IL-20R1						
+				10 (83)	8 (67)	10 (83)
-				**–**	**–**	**–**
***P*** **-value**				**–**	**–**	**–**
TNF-α						
+					7 (70)	9 (90)
-					1 (50)	1 (50)
***P*** **-value**					1.000	0.318
IL-1β						
+						6 (75)
-						4 (100)
***P*** **-value**						0.515

Data are presented as numbers (percentage) and compared using the Fisher exact test. * *P* < 0.05

### IL-20R1 and IL-20R2 were expressed in the disc tissues of patients with DLS

**Fig. 2:**
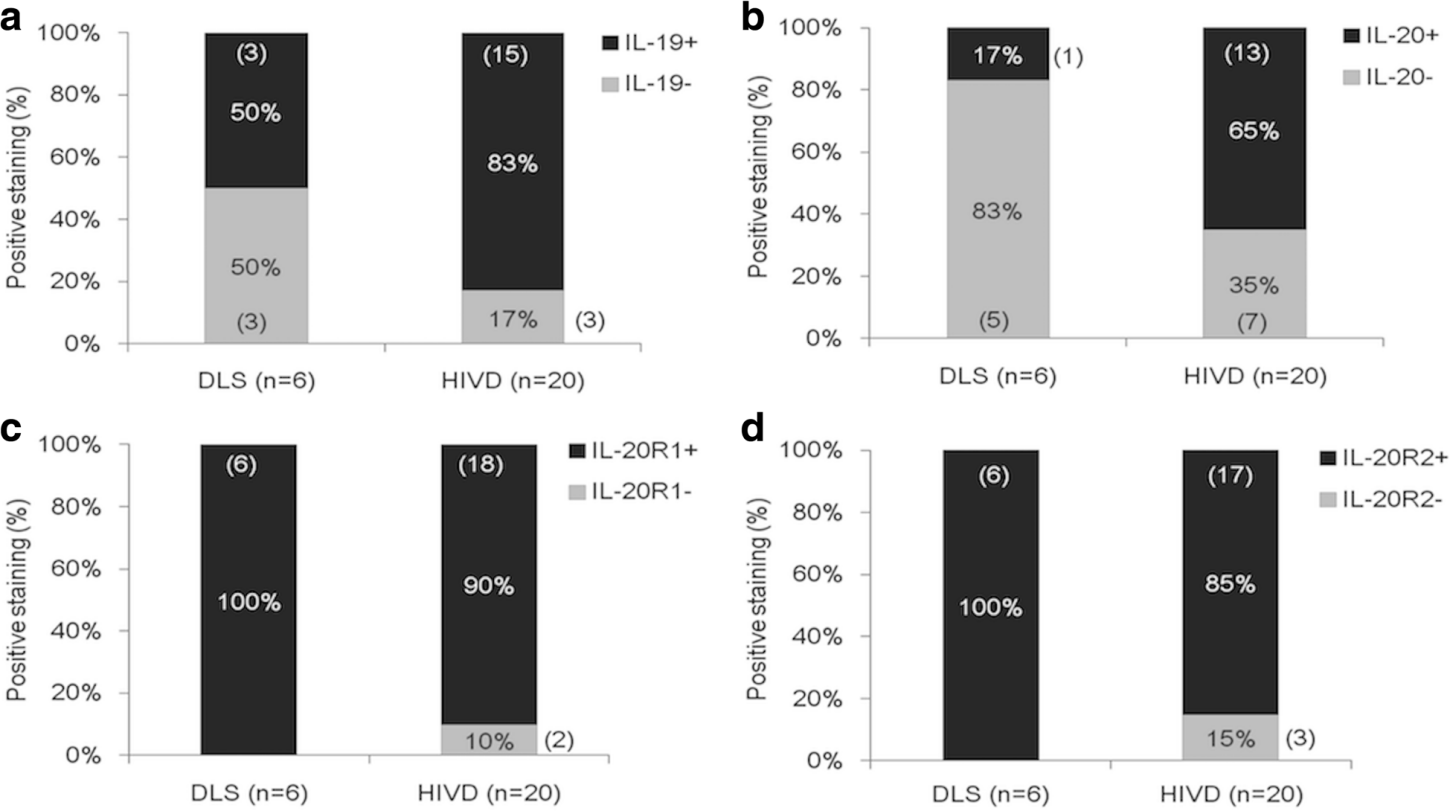
Expression of IL-19, IL-20, and their receptors in DLS patients and HIVD patients. Data comparison of positive staining results of IL-19, IL-20, IL-20R1, and IL-20R2 in disc tissues from DLS patients and HIVD patients. Expression of (**a**) IL-19, (**b**) IL-20, (**c**) IL-20R1, (**d**) IL-20R2 were analyzed by using IHC staining in disc tissues from DLS (*n* = 6) or HIVD (*n* = 20) patients (data of HIVD patients came from [22])

We previously showed that IL-20 and its receptors were expressed in human herniated intervertebral disc (HIVD) tissues [22]. In this study, we further compared the expression of IL-19, IL-20, and their receptors (IL-20R1 and IL-20R2) in the discs between elderly patients with DLS and adult patients with HIVD. It was of interest that the percentages of positive staining of IL-19 and IL-20 were higher in the discs from adult patients with HIVD (83% and 65%, respectively) than in those from elderly patients with DLS (50% and 17%, respectively). Besides, IL-20R1 and IL-20R2 were all expressed in disc tissues from DLS patients (100% and 100%, respectively) and from HIVD patients (90% and 85%, respectively, Fig. 2). These data indicated that disc cells might be the possible target cells for IL-19 and IL-20 involved in the pathogenesis of DLS and HIVD.

### IL-19 and IL-20 induced proinflammatory cytokine and chemokine expression in disc cells

**Fig. 3:**
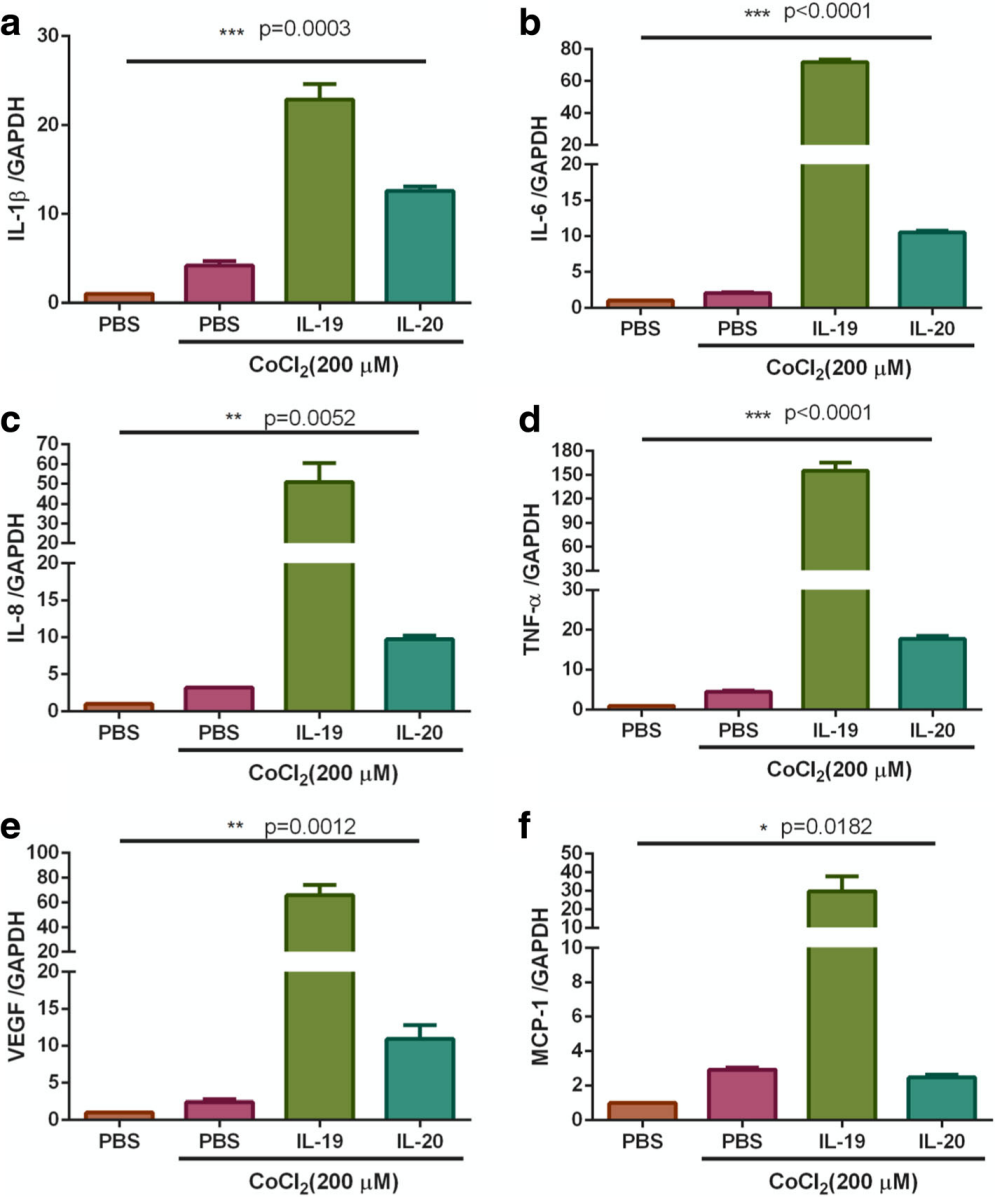
IL-19 and IL-20 induced cytokine and chemokine expression in DLS disc cells. Primary disc cells isolated from DLS patients were incubated with IL-19 or IL-20 in CoCl_2_-mimicked hypoxia condition. The expression of cytokines and chemokines was then analyzed using real-time PCR with primers specific for (**a**) IL-1β, (**b**) IL-6, (**c**) IL-8, (**d**) TNF-α, (**e**) VEGF, and (**f**) MCP-1. The relative quantification of PCR products was expressed as 2^−ΔΔCT^, normalized using GAPDH expression and relative to the levels of PBS-treated disc cells, data statistics were done by one way ANOVA, * *p* < 0.05; **: *p* < 0.01; ***: *p* < 0.001

Cartilage and disc tissues are hypoxic and avascular tissues. Previous studies indicated that hypoxia-inducible factors (HIF-1α and HIF-2α) are expressed in this tissue and play a role during intervertebral disc degeneration [10–12]. To mimic the pathological environment of degenerative lumbar spondylolisthesis, we used CoCl_2_-mimicked hypoxic conditions to increase HIF-α for in vitro culture system. To further clarify the role of IL-19 and IL-20 in DLS, we investigated whether IL-19 or IL-20 altered the in vitro expression levels of other proinflammatory cytokines and chemokines in the disc cells under CoCl_2_-mimicked hypoxic conditions. Real-time PCR showed that both IL-19 and IL-20 induced the expression of IL-1β, IL-6, IL-8, TNF-α, VEGF, and MCP-1 in isolated disc cells (Fig. 3). In addition, we observed that IL-19 had a stronger effect than IL-20 for inducing cytokine and chemokine expression in disc cells derived from DLS patients.

## Discussion

Degenerative lumbar spondylolisthesis (DLS), more common at the levels of L4–5, which characterized by degenerative arthritis of facet joints in associated with disc and ligamentum flavum degeneration, and presents as long history of back pain, sciatica and/or neurologic claudication [31]. The etiology of DLS is multi-factorial, but the role of IL-19 and IL-20 in the inflammation of DLS was simultaneously investigated in the three degenerated structures of disc, facet joint, and ligamentum flavum. We found that IL-19 and IL-20 were expressed and accompanied by abundant expression of TNF-α, IL-1β, and MCP-1 in the inflamed degenerated facet joints of patients with DLS. IL-19 and IL-20’s receptors (IL-20R1 and IL-20R2) were also expressed on chondrocytes and fibrocytes/fibroblasts in the disc, facet joint and ligamentum flavum tissues in patients with DLS. These data suggested that chondrocytes in the degenerative tissues of DLS, especially the facet joint, could be the target cells of IL-19 and IL-20. IL-19 and IL-20 might exert an autocrine response of the inflammatory process of facet joint in patients with DLS.

Previous study [32] indicated that the levels of IL-6, IL-7, IL-13, TNF-α, interferon-γ (IFN-γ), and platelet derived growth factor (PDGF) were increased in the subchondral facet joint of DLS patients due to the elevated activities of osteoclasts and osteoblasts, and the levels of IL-6, IL-8, and TNF-α were elevated in both annulus fibrosus (AF) and nucleus pulposus (NP) samples of DLS patients, but the anti-inflammatory IL-1 receptor antagonist (IL-1ra) decreased in NP, indicated the inflammation is ongoing in the disc of DLS patients, suggested that inflammation and remodeling might be the possible causative factors in the osteoarthritis of facet joint in DLS; and the intervertebral disc degeneration (IVDD) also participated in the development of DLS.

IL-20 had significant correlations with IL-1β expression in facet joint. Clinical studies [30, 33] have shown increased levels of IL-1β in human facet joint tissue from patients undergoing surgery for lumbar spinal stenosis and disc herniation. They also showed that the concentrations of IL-1β in the facet joint tissue of patients with lumbar spinal stenosis correlated with leg pain, and hypothesized that IL-1β leaks from facet joints to the nerve roots and thus induces radiating sciatic pain [33]. We found that there was a significant correlation between the expression of IL-20 and IL-1β in facet joint tissues of DLS patients. Whether the mechanism of leg pain is associated with the regulation between IL-20 and IL-1β in facet joint awaits further investigation. Therefore, IL-20 might be associated with the inflammatory reaction in facet joint of DLS, and play a more important role in facet joint than ligamentum flavum and disc in DLS. Based on the observation, the etiology of DLS might be more facet-centric than ligamentum flavum, or disc related in view of our results. Manipulation of IL-20 expression in the facet joint might be a possible mechanism for reducing inflammatory response in DLS patients.

Previous study [34] reported that IL-20 directly or indirectly promotes angiogenesis through VEGF. In the present study, we also found that both IL-19 and IL-20 all induced VEGF expression and upregulated another angiogenic factor, IL-8, in hypoxic disc cells, which suggested that IL-19 and IL-20 are involved not only in inflammation, but also might involve in the regulation of angiogenesis in the tissue of patients with DLS. We observed that IL-19 was expressed in a higher ratio in disc tissue samples than IL-20, which suggested that IL-19 might be a key factor in the degeneration of discs in patients with DLS. In addition, IL-19 was a more potent in vitro stimulator for proinflammatory cytokine expression than IL-20 for disc cells*.* However, it is not clear whether IL-19 is also more potent than IL-20 in vivo for disc degeneration. The implication of this finding should encourage further study.

We also compared the expression of IL-19 and IL-20, and their receptors in disc tissues between elderly patients with DLS and adult patients with HIVD and found that the frequency of IL-19 and IL-20 expression was higher in the disc tissues of HIVD than DLS, but the expression of their receptors was all expressed in HIVD and DLS. It may be due to the immune system of the young HIVD patients were strong and active, while the immune system of the elder patients with DLS were weak and immunosenescence. The frequency of expression of IL-19 and IL-20 in the disc tissues of young patients with HIVD was higher than in elderly patients with DLS. Therefore, we speculated that the inflammatory reaction was more severe in herniated disc tissues of young adults with HIVD than degenerative disc tissue of elderly patients with DLS. The immune property of nucleus pulposus might play an important role in the autoimmune and acute inflammation in younger patient with HIVD, while the inflammation in elderly patients with DLS tend to be chronic and repetitive with a smaller content and more degeneration of nucleus pulposus.

IL-19 and IL-20 upregulated the expression of TNF-α, IL-1β, IL-6, IL-8, VEGF, and MCP-1 in disc cells isolated from DLS patients under CoCl_2_-mimicked hypoxic conditions, provide another evidence to support our hypothesis that IL-19 and IL-20 might contribute to the inflammatory response, angiogenesis, and chemotaxis in disc cells after DLS. IL-19, IL-20 and their receptors may be important generators of inflammation in degenerated disc tissues of DLS.

We studied 13 cases of DLS and analyzed several kinds of inflammatory change of disc, facet joint, ligamentum flavum, and discussing the specimen obtained from surgical intervention. This is a pilot study to investigate the role of inflammation in the three different tissues of DLS, although there have been some intriguing findings, but the small number of cases is limitation in this study, and need large-scale future study to support the findings. Targeting proinflammatory cytokines may provide novel and effective strategy for patients with DLS by blocking DLS-related inflammation and reducing the progression of the disease.

## Conclusion

In this study, our data suggests that IL-19 or IL-20 may be an initiator of the inflammatory response in DLS. IL-19, IL-20, and their receptors as well as proinflammatory cytokines were expressed more frequently in facet joint than ligamentum flavumand disc in patients with DLS. IL-19 and IL-20 induced proinflammatory cytokine expression in disc cells of DLS. Therefore, the inflammatory response might be more facet-centric in DLS. IL-19 or IL-20 might play a role in the pathogenesis of DLS.
